# Prognostic Significance of Glutathione Peroxidase Levels (GPx1) in Head and Neck Cancers

**DOI:** 10.3389/fonc.2017.00084

**Published:** 2017-05-09

**Authors:** Didier Dequanter, Ruveyda Dok, Louet Koolen, Vincent Vander Poorten, Sandra Nuyts

**Affiliations:** ^1^Laboratory of Experimental Radiotherapy, Department of Oncology, KU Leuven, Belgium; ^2^Department of Head and Neck Surgery, CHU Saint Pierre, Brussels, Belgium; ^3^Otorhinolaryngology, Head and Neck Surgery, Department of Oncology, Section Head and Neck Oncology, University Hospitals Leuven, KU Leuven, Leuven, Belgium; ^4^Department of Radiation Oncology, Leuven Cancer Institute, University Hospitals Leuven, Leuven, Belgium

**Keywords:** oxidative stress status, head and neck cancer, glutathione peroxidase, prognostic significance

## Abstract

**Introduction:**

To date, no reliable prognostic biological marker for all squamous cell carcinoma located in different subsites of the head and neck region has been identified and used in daily routine. In line with our previous studies, in which we showed a role of glutathione and associated enzymes as potential biological markers, we investigated the relationship between GPx1 and prognosis of head and neck squamous cell carcinoma.

**Methods:**

The association between GPx1 and patient and tumor related factors were investigated in 87 pretreatment biopsies from head and neck cancer patients treated by (chemo)radiation. Moreover, the influence of GPx1 expression on outcome parameters was assessed.

**Results:**

A significant difference was found in the T-stage between the low and high-expressing GPx1 groups. About 75% of the T3–T4 tumors were considered GPx1 low-expressing tumors, while low GPx1 expression was only seen in 25% of the T1–T2 tumors. There was also a significant difference found between the groups when looking at the different tumor sites. Local control, locoregional control, disease-free survival, and overall survival were the same in both groups. All these results indicate that GPx1 expression does not influence the radiotherapy response nor survival.

## Introduction

Cancer of the head and neck region represent the fifth most common form of cancer and caused 350,000 deaths per year (1, 2). Tumors of the head and neck have strong links to oxidative damage and oxidative stress, with tobacco and alcohol clearly defined as major etiologic factors.

So far, no reliable prognostic biological marker for the squamous cell carcinoma located in different subsites of the head and neck region has been identified and used in daily routine. In accordance with other authors, we believe that potential markers can be found among the parameters of oxidative stress (3, 4).

Therefore, in line with our previous studies pointing at the role of glutathione and associated enzymes as potential biological markers (5, 6), we have investigated relationships between GPx1 and prognosis of head and neck squamous cell carcinoma (HNSCC).

By reducing hydrogen peroxide at the expense of oxidizing GSH to its disulfide form, GSSG, GPx1 is a major enzyme of the cellular antioxidant armada (3). It is present in cytosolic and mitochondrial compartments, but, in some cells, in peroxisomal compartments (7). GPx1 has been found to be more effective than catalase at protecting the cells again intracellular peroxides under many physiological conditions.

GPx1 activity is often compared with glutathione reductase activity, which role is to maintain a constant level of GSH from GSSG for enzyme activity. Furthermore, GPx1 is highly upregulated during oxidative stress making it a suitable biomarker for oxidative stress (8). Therefore, in this retrospective study, we investigated the prognostic value of GPx1 expression in head and neck cancers.

## Materials and Methods

### Study Cohort

The study cohort consisted out of 87 HNSCC patients (73 males, 14 females, median age 58.2 years) who underwent definitive radiotherapy or concomitant chemoradiotherapy as primary oncological treatment at the University Hospital in Leuven. Treatment modality was based on the extent of the disease at initial presentation and performance status of the patient. Radiotherapy alone was initiated in 31 patients (36%). All patients received 72 Gy of radiation; treatment was administered once a day, five times a week the first 4 weeks of therapy, the last 2 weeks two fractions per day were administered (9). Forty-nine of the 87 patients (56%) received concomitant chemoherapy based on cisplatin 100 mg/m^2^ week 1 and week 4. Six patients (7%) were treated by radiotherapy with concurrent cetuximab. Cetuximab was chosen in those patients with contraindications for cisplatin. Median follow-up was 5.54 years (4.15–7.67).

The study was performed according to protocols approved by the Ethical board of the University Hospitals (Commissie Medische Ethiek van de Universitaire ziekenhuizen KULEUVEN), and all the patients provided inform consent. Histological examination of the tumor biopsies indicated squamous cell carcinomas in all cases. Human papillomavirus (HPV) and p16 status was determined as previously described (10).

### Immunohistochemistry of Tumor Biopsies

For immunostaining of GPx1 (HPA044758; Sigma-Aldrich), routine formalin-fixed paraffin-embedded (FFPE) pretreatment tumor tissues, available for 87 HNSCC patients were used. Briefly, 4-µm FFPE tumor sections were deparaffinized in Ultraclear and rehydrated in 100% ethanol. Endogenous peroxidases were blocked by using 0.3% H_2_O_2_ in Methanol solution. Heat-induced antigen retrieval (pH 6) was performed. Immunodetection was performed with EnVision HRP Anti-Rabbit secondary antibody and peroxidase/DAB kit (Dako). Sections were counterstained with hematoxylin. Scoring of GPx1 was performed by multiplying the intensity (Table 1) and percentage of tumor cells (Table 2). High GPx1-expressing tumors were defined by a high-intensity score (3) and high-scoring categories (>3) according to the percentage of GPx1 positive tumoral cells (>50%).

**Table 1:** **Scoring categories according to the intensity of GPx1 labeling in the tumor**.

Intensity	Score
Low	1
Medium	2
High	3

**Table 2:** **Scoring categories according to the percentages of GPx1 positive cells in the tumor**.

Percentage of stained cells	Scoring
<10	0
10–25	1
25–50	2
50–75	3
>75	4

### Statistical Analysis

Differences in GPx1 high- and low-expression groups were analyzed using the Chi-square test in case of categorical predictors, whereas one-way ANOVA was used in cases of continuous predictors. Former smokers are defined as patients who stopped smoking longer than 1 year prior to the date of diagnosis. Local control (LC) and locoregional control (LRC) were defined as the time between the start of treatment and the date of local or locoregional recurrence. Time between the start of treatment and disease recurrence or death expressed the disease-free survival (DFS). Overall survival (OS) was determinated by the time between the initial treatment and death of any cause. The survival curves were generated and calculated by using, respectively, the Kaplan–Meier method and the log-rank test. All analyses were performed using the Statistica software version 12, and all test were considered significant when *p* ≤ 0.05.

## Results

**Figure 1:**
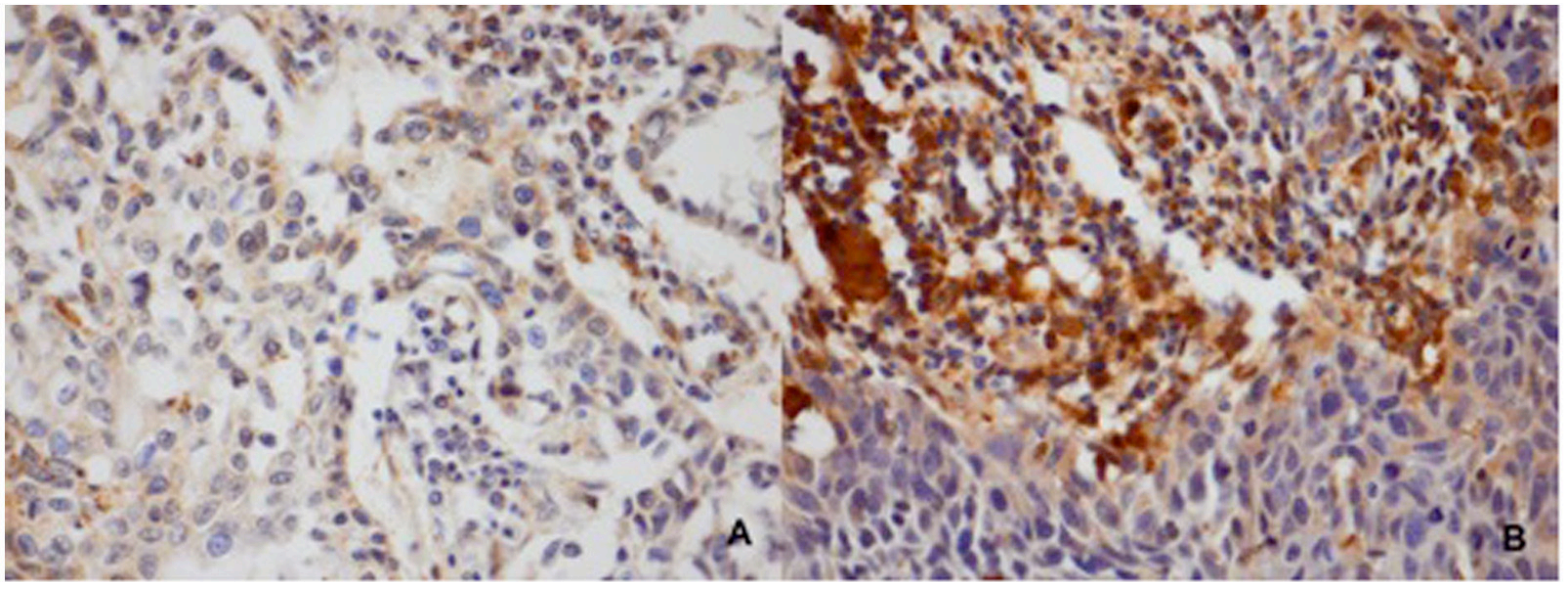
**Classification of the GPx1 staining according to intensity and the percentage of stained cells**. **(A)** Tumors classified as low levels of GPx1. **(B)** Tumor classified as high levels of GPx1.

A total of 87 pretreatment patient tumor samples were stained for GPx1 (Figure 1). The baseline patient and tumor characteristics according to GPx1 expression are summarized in Table 3. Twenty-eight percent of the total amount of patients (24 out of 87) showed a high expression of GPx1. The majority of patients (72%) showed low expression levels of GPx1. The age, treatment modality, and HPV/p16 status did not differ between the two different groups. No relation was found between GPx1 expression and smoking history. Also, no significant differences were found in nodal status between the two groups. However, a significant negative correlation between T-status and GPx1 (*r* = −0.23; *p* = 0.028) was noted. About 75% of the T3–T4 tumors were considered GPx1 low-expressing tumors, while low GPx1 expression was only seen in 25% of the T1–T2 tumors. Also, significant differences were found between the GPx1 groups and different tumor sites.

**Table 3:** **Patient and tumor characteristics by GPx1 expression**.

Data	GPx1 high		GPx1 low		All patients		*p*-Value
No of patients	No.	%	No.	%	No.	%	
	24	28	63	72	87		
Gender							NS
Male	19	79	54	86	73	72	
Female	5	21	9	14	14	28	
Age, years							NS
Median (range)	58.6 (46–76)		58.0 (43–78)		58.2 (43–78)		
Nodal status							NS
N0/N1	8	33	25	40	33	38	
N2/N3	16	67	38	60	54	62	
Tumor status							*p* = 0.028
T1/T2	12	50	16	25	28	32	
T3/T4	12	50	47	75	59	68	
Disease stage							NS
I–II	1	4	2	3	3	3	
III–IV	23	96	61	97	84	97	
Tumor site							*p* = 0.033
Soft palate	1	4	2	3	3	3	
Tonsil	8	33	19	30	27	30	
BOT/vallecula	14	59	19	30	33	37	
Pharyngeal wall	0	0	9	15	9	14	
Unknown	1	4	14	22	15	16	
Human papillomavirus (HPV)							NS
HPV negative	15	63	35	56	50	57	
HPV positive	5	21	8	13	13	15	
Unknown	4	16	20	31	24	28	
Treatment							NS
RT	8	33	23	36	31	36	
RT + cetuximab	1	4	5	8	6	7	
RT + CT	15	63	34	54	49	56	
Unknown	0	0	1	2	1	1	
Smoking history							NS
Never	4	17	4	6	8	9	
Former	5	21	11	17	16	18	
Current	12	50	45	72	57	66	
Unknown	3	12	3	5	6	7	
p16							NS
Negative	3	12	19	30	22	25	
Nuclear	7	29	18	29	25	29	
Cytoplasmic	10	42	15	24	25	29	
Unknown	4	17	11	17	15	17	

*NS, not significant; BOT, base of tongue; RT, radiotherapy; CT, chemotherapy*.

**Figure 2:**
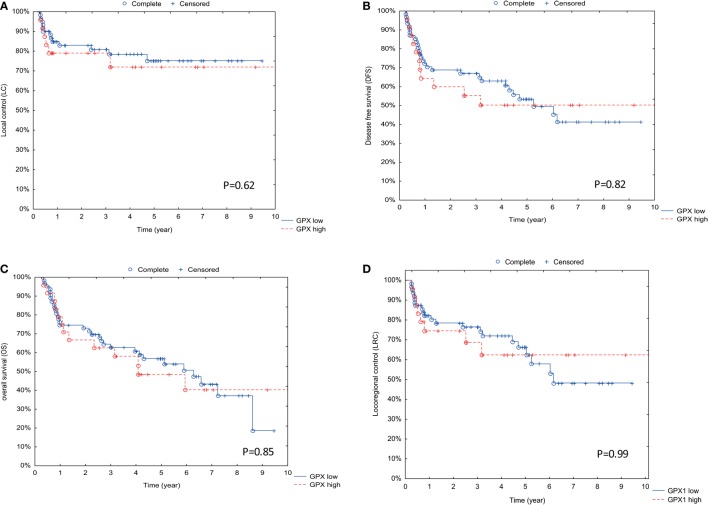
**Association between GPx1 expression and outcome parameters**. **(A)** Correlation between GPx1 expression and outcome with local control (LC) as endpoint. On the *y*-axis is the percentage of patients with LC displayed, and on the *x*-axis is the time in years displayed. **(B)** Correlation between GPx1 expression and outcome with disease-free survival (DFS) as endpoint. On the *y*-axis is the percentage of patients with DFS displayed, and on the *x*-axis is the time in years displayed. **(C)** Correlation between GPx1 expression and outcome with overall survival (OS) as endpoint. **(D)** Correlation between GPx1 expression and outcome with locoregional control as endpoint. On the *y*-axis is the percentage of patients with OS displayed, and on the *x*-axis is the time in years displayed. *p* Values are determined by log-rank tests.

Assessment of GPx1 expression and outcome parameters such as LC (Figure 2A), DFS (Figure 2B), OS (Figure 2C), and LRC (Figure 2D) did not result in significant differences.

All these results indicate that GPx1 expression does not influence the radiotherapy response nor the survival.

## Discussion

Cancer of the head and neck region and their treatment protocols still lack well-established prognostic biological markers (11). It is well known that non-HPV-related oropharyngeal cancers and HPV-related cancers are distinct entities concerning tumor biology and clinical outcome. For oropharyngeal cancers, HPV is a well-established prognostic marker to radiotherapy response (10) and survival. For non-oropharyngeal cancers, the HPV status is not of prognostic significance (12, 13) and, currently, there is no general guidance to adapt treatment strategies to HPV status. In line with previous publications, we believe that parameters of oxidative stress could offer useful potential markers (3, 4). Previously, we confirmed this hypothesis by demonstrating that a significant redox imbalance in head and neck cancer patients could offer a prognostic value (14).

Moreover, we reported a strong correlation between the contents of oxidized and reduced glutathione (GSSG/GSH) in tumors and the nodal status of our head and neck patients. A lower ratio GSSG/GSH was observed in tumor tissue of N0 patients, while a higher ratio GSSG/GSH was determined in positive node patients. This might suggest that high GSSH/GSH ratio tumors have a more aggressive phenotype caused by the oxidative stress, leading to a tendency to more local spread. Furthermore, compared to patients who showed high levels of oxidative stress ratio GSSH/GSH, patients with lower ratio GSSG/GSH showed lower risk of locoregional recurrence of their tumor after treatment. This again suggests that tumors with high-oxidative stress status are more aggressive.

These data lead us to extent our research by checking other components of the cellular antioxidant armada. In line with previous studies pointing to the role of glutathione-associated enzymes as potential markers, we have investigated relationships between GPx1 and prognosis of HNSCC.

Based on preclinical and clinical studies on glutathione peroxidase levels in tumors (15, 16), glutathione peroxidase, an important member of the defensive machinery against oxidative stress, was investigated.

No correlation was found between HPV status and the expression of GPx1. Furthermore, p16 status and localization did not differ between the groups of treated patients. A possible explanation could be the confounding effect of T-status on outcome. Our results contrast with the results presented by other authors. Williams et al. (17) suggested, in their study, that high risk types of human papillomavirus increased the level of reactive oxygen species, associated with a decrease of antioxidant enzyme GPx1 expression. This increased oxidative stress led to higher levels of DNA damage.

Furthermore, in contrast with our previous results, no significant correlation was found between the tumors expressing GPx1, found to be more effective than catalase at removing intracellular peroxides under many physiological conditions, and the nodal status. By contrast, Han et al. (18) found that high GPx1-expressing tumors were associated with extensive lymph node metastasis.

However, a significant correlation between T-status and GPx1 expression in tumors (*p* = 0.03) was found. The patients with tumors showing a low GPx1 expression had more tumors staged T3–T4. Moreover, we observed a significant correlation between tumors expressing GPx1 and the tumor localization. The patients with low GPx1-expressing tumors had more tumors located at the tonsil.

The function of GPx1 is to promote migration, proliferation, and tumor cell invasion, conditioning a potential not yet defined prognostic role, in cancer patients (19). The results published by Han et al support these findings, showing that expression of GPx1 was related to good outcome (*p* = 0.03) in patients with human gastric adenocarcinoma.

In hepatocellular carcinoma, Zmorzynski et al. (20) found that high GPx1-expressing tumors were correlated with a shorter survival time as in patients with prostate cancer.

Moreover, high GPx1-expressing tumors could be responsible for cisplatin resistance as observed in esophageal cancer cell lines (21). Similarly, Zhao et al. observed that GPx1 may serve as a molecular marker for monitoring bladder cancer recurrence (22). In patients with gastric cancer, low expressed GPx1 tumors were associated with aggressiveness and poor survival (23).

Interestingly, our results suggest the absence of the prognostic value of GPx1 expression in head and neck cancers. No significant differences were found in any of the other characteristics: LC, LRC, DFS, and OS. A possible explanation would be that a small biopsy of the tumor does not represent the whole tumor, taking into account the issue of tumor heterogeneity. Moreover, since, we only had the availability of small paraffin-embedded biopsies, only one immunohistochemistry staining for GPx1 could be performed. Several other markers of the oxidative stress pathway could be investigated (24). New research into others markers of oxidative stress seems valuable.

To determine the cellular antioxidant capacity, next to other isoforms of glutathione peroxidase as GPx4, the expression of other important antioxidant enzymes, such as manganese superoxide dismutase and its isoform SOD2 will be of interest as proven in preliminary animal studies (25).

To elucidate the potential clinical significance of GPx1 observed in other solid tumors, large studies, analyzing multiple glutathione-associated enzymes in addition to GPx1, are needed.

## Conclusion

After determining the levels of oxidative stress in 87 HNSCC patients, significant clinical differences were found between patients expressing high level of GPx1 and patients expressing low levels of GPx1. A significant negative correlation between T-stage and GPx1 expression in tumors (*p* = 0.03) was found. The patients with tumors showing a low GPx1 expression had more tumors staged T3–T4. Moreover, we observed a significant correlation between tumors expressing GPx1 and the tumor localization. The patients with low-expressing GPx1 tumors had more tumors located at the tonsil. Interestingly, no significant differences were found in any of the outcome characteristics: LC, LRC, DFS, and OS, suggesting the absence of the prognostic value of GPx1 expression is HNSCC.

## Author Contributions

DD, RD, LK, and SN contributed equally to the redcation of the manuscript.

## Conflict of Interest Statement

The authors declare that the research was conducted in the absence of any commercial or financial relationships that could be construed as a potential conflict of interest.
